# Machine learning decision tree models for multiclass classification of prognosis in patient undergoing palliative radiotherapy for bone metastases

**DOI:** 10.1002/acm2.70606

**Published:** 2026-07-08

**Authors:** Savino Cilla, Romina Rossi, Ragnhild Habberstad, Pal Klepstad, Monia Dall Agata, Stein Kaasa, Vanessa Valenti, Federica Medici, Costanza M. Donati, Marco Maltoni, Alessio G. Morganti

**Affiliations:** ^1^ Medical Physics Unit Responsible Research Hospital Campobasso Italy; ^2^ Palliative Care Unit IRCCS Istituto Romagnolo Studio Tumori “Dino Amadori” Meldola Italy; ^3^ Department of Clinical and Molecular Medicine Norwegian University of Science and Technology Trondheim Norway; ^4^ Department of Oncology St. Olavs University Hospital Trondheim Norway; ^5^ Department of Circulation and Medical Imaging Norwegian University of Science and Technology Trondheim Norway; ^6^ Department of Anaesthesiology and Intensive Care Medicine St Olavs University Hospital Trondheim Norway; ^7^ Unit of Biostatistics and Clinical Trials IRCCS Istituto Romagnolo per lo Studio dei Tumori (IRST) “Dino Amadori” Meldola Italy; ^8^ Department of Oncology Oslo University Hospital Oslo Norway; ^9^ Radiation Oncology, Department of Medical and Surgical Sciences (DIMEC) Alma Mater Studiorum University of Bologna Bologna Italy; ^10^ Medical Oncology Unit, Department of Medical and Surgical Sciences (DIMEC) Alma Mater Studiorum‐University of Bologna Bologna Italy; ^11^ Radiation Oncology IRCCS Azienda Ospedaliero‐Universitaria di Bologna Bologna Italy

**Keywords:** artificial intelligence, life expectancy, machine learning, palliative radiotherapy, predictive models, prognosis, survival

## Abstract

**Background:**

Accurate estimation of prognosis and life expectancy is essential in patients with advanced cancer, as it guides clinical decision‐making and helps avoid unnecessary interventions while facilitating timely integration of palliative and supportive care. Palliative radiotherapy plays a key role within multidisciplinary management, offering effective and well‐tolerated symptom relief for complications such as pain, bleeding, and obstruction, with treatment strategies closely tailored to expected survival. Although recent advances in machine learning have improved prognostic accuracy by modeling complex variable interactions, their application in palliative care settings remains limited.

**Purpose:**

To aid clinical decision‐making, we developed a decision tree multi‐classifier to predict the mortality at 3, 24, and 52 weeks following palliative radiotherapy for bone metastases.

**Methods:**

Data from 573 adults diagnosed with metastatic cancer were analyzed. The primary endpoint was the overall survival (OS) defined as the number of months from treatment to death event. Four clinically relevant classes were defined: Class 0 (OS: ≤ 3 weeks), Class 1 (OS: 3–24 weeks), Class 2 (OS: 24–52 weeks) and Class 3 (OS ≥ 52 weeks). Candidate covariate predictors consisted of 65 clinical, dosimetric and laboratory variables. Two supervised decision tree machine‐learning models were trained and validated using the Python package. A SHapley Additive exPlanations (SHAP) explanaibility analysis was performed to infer the global and local feature importance.

**Results:**

The SHAP analysis selected three laboratory variables, the interleukin8, haemoglobin and lymphocytes count as the first three ranked variables representing the major impact on OS in each of the four classes and accounting for more than 80% of contribution. In all classes, higher chance of OS was associated with low values of interleukin8 (IL8) and higher values of haemoglobin (HEM) and lymphocytes count (LYMPH). Pre‐treatment values of IL8 > 36.7 relocated more than 50% of patients with survival < 3 weeks and only 1.5% of patient with survival > 52 weeks. On the other hand, pre‐treatment values of IL8 < 19 relocated about 92% of patients with survival > 52 weeks. Patients are then additionally separated based on the lymphocytes count (LYMPH). LYMPH values higher than 7.5 will drive the probability of survival > 52 weeks still over 90% while it drops down to 2.1% for LYMPH < 7.5.

**Conclusion:**

An explainable machine learning approach based on decision trees is able to predict the survival at different timing after radiotherapy in patients with advanced cancer. This approach provides an intelligible explanation of individualized risk prediction, helping clinicians to identify the best strategy for patient stratification and treatment selection.

## 1 INTRODUCTION

A reliable evaluation of prognosis and life expectancy is essential for patients with advanced cancer. The ability to anticipate the need for palliative and supportive therapies can help prevent unnecessary medical procedures. Palliative radiotherapy (PRT) is a critical component of multidisciplinary management, representing a well‐tolerated, effective treatment for pain, bleeding, obstruction, and other symptoms/complications of advanced cancer.^1^ Because the purpose of treatment is greatly influenced by life expectancy, radiation oncologists are aware that a precise prognosis is a crucial consideration when choosing the best radiation therapy regimen.^2^

In recent years, the advances in machine learning (ML) algorithms, which are able to model linear and nonlinear interactions among a great number of variables, allowed for more accurate cancer prognoses.^3^, ^4^ Nevertheless, advances in machine learning have not yet significantly impacted palliative care.^5^ Nowadays, only two research studies using ML models for outcome prediction PRT have been reported. The bone metastases ensemble trees for survival (BMETS) model was developed with the aim to predict overall survival (OS) in patients PRT for bone metastases, reporting excellent discrimination on external validation.^6^, ^7^ Another model, based on more than 4000 predictor variables, was developed by the Stanford group with the aim to predict survival in patients receiving PRT.^8^ However, because of the model's complexity, its applicability to external users has not yet been established.

The absence of accurate predictive models constitutes a real obstacle to the delivery of tailored treatment for patients with advanced cancer, particularly in view of the recent discoveries of molecular, biological, and clinical factors with potential prognostic value.^9^, ^10^ Recently, a longitudinal observational multicentric study on 600 patients with cancer‐induced bone pain receiving PRT was carried out to identify potential biomarker predictors for pain relief (PRAIS Trial).^11^ According to the authors, higher performance status, the use of corticosteroid, and soft tissue expansion outside of bone were all found to be predictive of PRT response.^12^ Based on the data of the aforementioned PRAIS trial, we recently performed a preliminary analysis to combine a ML model with explainable artificial intelligence to predict 1‐year survival after PRT for bone metastasis.^13^ The results provided further evidence that, in addition to clinical variables, laboratory‐based data also have a strong prognostic significance in survival prediction after PRT.

However, most of studies focused on predictive models are based on dichotomized classifications of clinical outcomes. Machine learning decision‐tree (DT) are one of the most powerful data mining methods used for multiclass classifications,^14^ closer to the clinician reasoning in the daily clinical practice. This is a non‐parametric and nonlinear algorithm, providing easy and explainable predictive models where the hierarchical relationships between the target variables and the covariates are presented in tree‐like hierarchical structures to mine the classification rules. In the field of PRT, this approach is particularly attractive, being able to investigate the complex relationships between clinical, biological, tumur and treatment‐related variables and their impact on different survival endpoints.

The present study is a secondary analysis of the aforementioned PRAIS trial.^11^ Based on this large database of clinical, tumor and treatment‐related and laboratory data, we hypothesize that DT algorithms have the potential to provide a multiclass prediction of mortality risk at different timing after PRT treatment. We developed and validated DT multi‐classifiers to predict mortality at 3, 24, and 52 weeks following PRT for bone metastasis, and investigated the underlying processes involved in the hierarchization of the decision‐making algorithms of the models.

## 2 Material and Methods

### 2.1 Study cohort

The present study is a secondary analysis of the international longitudinal observational Palliative Radiotherapy and Inflammation Study (PRAIS),^11^ whose primary aim was the identification of potential predictors for pain relief from PRT. Candidate covariate predictors consisted of 65 variables, including 7 clinical pre‐PRT patient characteristics, 6 tumors‐related variables, 7 dosimetric and treatment‐related variables, and 45 laboratory variables. An overall overview of the collected variables is reported in the Table S1 in supplementary materials. The final cohort evaluable for statistical analysis in the present study comprised 573 patients.

### 2.2 Endpoints definition

The target variable, that is, the patients’ OS, was defined as the number of months from treatment to death event. For the classification purposes, four clinically relevant classes were defined as: Class 0 (OS: ≤ 3 weeks), Class 1 (OS: 3–24 weeks), Class 2 (OS: 24–52 weeks) and Class 3 (OS ≥ 52 weeks).

### 2.3 Decision tree algorithms

Decision trees (DT) are supervised machine‐learning models used to solve classification and regression problems.^14^ They are tree‐like structures, where each internal node tests on attribute, each branch corresponds to attribute value and each leaf node represents the final decision or prediction. The process of forming a decision tree involves recursively partitioning the data into subsets based on the values of different attributes, by choosing a variable at each step that best splits the set of items. The best splits are obtained by different metrics by different algorithms, typically gauging the target variable's homogeneity within the subsets. A quality score for the split is obtained by applying these metrics to each candidate subset and combining (e.g., averaging) the yielding values. A first approach uses the concepts of entropy (E) and information gain (IG), evaluating, at each decision node, the ‘purity’ of data as:

$$E=-\sum_{1}^{n}p_{i}log_{2}\left(p_{i}\right)$$

where the p_i_ is the probability of randomly selecting an example in class i. Entropy basically quantifies the data's randomness. The aim is to minimize this entropy, in order to obtain subsets as homogeneous as possible. The Information Gain (IG)—the reduction in entropy attained as a result of the split—is computed by the decision tree to determine which feature to split over. In details, the algorithm determines a potential split for each variable, then computes the average entropy for either or both nodes, and lastly computes the entropy change with respect to the parent node. This way, IG represents how much information a feature provides for the target variable.

$$IG=E_{parent}\;-\sum_{1}^{k}E_{j,son}$$

The feature with the highest IG is chosen for the split. This procedure keeps going until the data is sufficiently pure or the tree reaches a specific depth.

A second approach uses the concept of Gini impurity (GI) or Gini's diversity index. Gini impurity quantifies the likelihood that an element selected at random from a set will be mislabelled if the labels are applied randomly and independently based on the distribution of labels in the set. When every case in the node fits into a single target category, it reaches its minimum (zero). For a set of items with 𝐽 classes and relative frequencies pi, i Є {1,2,….,J}, the probability of choosing an item with label *i* is *pi*, and the probability of miscategorising that item is:

$$\;\sum_{k\neq i}p_{k}=1-p_{i}$$

The Gini impurity is then computed by summing pairwise products of these probabilities for each class label:

$$\begin{array}{cc} GI & =\sum_{i=1}^{J}\left(p_{i}\sum_{k\neq i}p_{k}\right)\\ & =\sum_{i=1}^{J}p_{i}\left(1-p_{i}\right)=\sum_{i=1}^{J}\left(p_{i}-p_{i}^{2}\right)=1-\sum_{i=1}^{J}p_{i}^{2} \end{array}$$

The primary objective of the Gini index is to differentiate the majority of categories within the data from other categories at various nodes. A lower Gini value indicates a more uneven category distribution within the sample. This implies that a higher category purity in the subset produced by the splitting point improves the capacity to discriminate between various categories.

### 2.4 Preprocessing, variable importance and selection

To meet preprocessing requirements for machine learning, highly correlated features were removed using a threshold of 0.85 in Pearson correlation for continuous and Spearman correlation for categorical features. Missing data were assumed missing at random and non‐dependent on the outcome. Missing data was imputed using the multiple imputations with chained equations (MICE) strategy.

The evaluation of variable importance and selection is a relevant issue within machine learning modeling. This issue focuses on the selection of a smaller subset of variables representing the overall data but eliminating the redundant and irrelevant information. In this paper we used two different variable selection methods: a) the commonly used importance‐based method, embedded within the decision tree algorithms and b) the Shapley Additive exPlanation (SHAP)‐value‐based selection. Variable importance is a measure of how much each variable contributes to the overall accuracy of the decision tree model. In decision tree models, it is calculated based on the reduction in impurity or error, measured by entropy or Gini impurity, that each feature brings when it is used to split a node. In Python, the feature importance can be easily computed and visualized using the scikit‐learn library, implementing the so‐called “feature importances” attribute of the fitted decision tree model and getting an array of feature importance values. On the other side, the SHAP based selection method is based on the calculation of Shapley values, a game theory concept that explains the importance of an individual player (a variable) in a collaborative team (the set of all variables). Specifically, the Shapley value for a variable is defined as the average marginal contribution over all possible coalitions. The objective of this game would be to predict an instance of the dataset; the gain would be the discrepancy between the prediction and the average prediction of all instances; and the players would be the values of the various variables collaborating together to obtain the prediction. By identifying the traits that have a greater or lesser influence on a model's predictions, Shapley values enable to quantify the impact that every variable has on those predictions. Given that the Shapley value can be utilized to quantify the impact of a feature on a prediction, the mean of the absolute values of the Shapley values for each feature's observations can be used to determine the overall impact of a variable. This metric can be used to prioritize the variables and create a ranking.

### 2.5 Decision trees modeling

Multi‐class classification of imbalanced data represents a major significant challenge, especially in predicting minority class examples due to the skewed distribution of output classes. Suitable optimization strategies must be undertaken to prevent over fitting and to address the bias‐variance trade‐off. This involves tuning the hyperparameters of the machine learning algorithms and implementing an appropriate cross‐validation mechanism. In the present work, DT algorithms are developed in the Python scripting language using the Scikit‐learn library. The hyperparameters of both models were optimized using the GridSearchCV routine. Moreover, in order to consider the classes unbalanced, the splitting criteria of the tree have been updated to not only take the purity of the split but also be weighted by the importance of each class. The scikit‐learn Python library provides an implementation of the decision tree algorithm that supports class weighting, called the *class_weight* argument that can be specified as a model hyperparameter.

A hold‐out split dataset strategy was implemented, using 80% and 20% of the data for training and testing, respectively. In addition, a five‐fold cross‐validation was performed to further reduce the risk of overfitting and to evaluate the performance of the developed models.

### 2.6 Statistical analysis

For continuous and categorical variables, mean values and frequencies were expressed with their standard deviations (± SDs) and percentages (%), respectively. The normal distribution of continuous variables was assessed with a Kolmogorov–Smirnov test. The differences among the four classes were tested using the one‐way ANOVA and Fisher post hoc tests.

The effectiveness of the DT models was evaluated in terms of the area under the receiving operating characteristic curve (AUC), precision, and recall. For multiclass classification purpose we choose the one‐vs.‐the‐rest strategy, computing a ROC curve per each of the 4 classes. In each step, a given class is regarded as the positive class and the remaining classes are regarded as the negative class as a bulk. Precision is the proportion of positive identifications that were actually correct; recall is the proportion of the actual positives that were correctly identified. However, in multi‐class classification these metrics need to be tweaked to measure the performance of each class and two commonly used measures of performance are used, namely the micro and macro averages. Micro average is calculated by taking the total number of true positives (TP), false positives (FP), true negatives (TN), and false negatives (FN) and then using these counts to calculate the precision, recall, and F1‐score as follows:

$$\;Precision_{micro}=\frac{\sum_{i=1}^{n}{\left(TP\right)}_{i}}{\sum_{i=1}^{n}{\left(TP\right)}_{i}+{\left(FP\right)}_{i}}$$

$$\;Recall_{micro}=\frac{\sum_{i=1}^{n}{\left(TP\right)}_{i}}{\sum_{i=1}^{n}{\left(TP\right)}_{i}+{\left(FN\right)}_{i}}$$

Micro average gives more weight to the majority class and is particularly useful when the classes are imbalanced.

Macro average, on the other hand, calculates the performance of each class individually and then takes the unweighted mean of the class‐wise performance, as follows:

$$\;Precision_{macro}=\frac{1}{n}\;\sum_{i=1}^{n}\frac{{\left(TP\right)}_{i}}{{\left(TP\right)}_{i}+{\left(FP\right)}_{i}}$$

$$\;Recall_{macro}=\frac{1}{n}\;\sum_{i=1}^{n}\frac{{\left(TP\right)}_{i}}{{\left(TP\right)}_{i}+{\left(FN\right)}_{i}}$$

Macro average gives equal weight to each class and is useful when all classes are of equal importance.

All statistical analysis, including machine learning training and testing, was performed using the Python v3.8 (Python Software Foundation, OR, USA) packages scikit‐learn, imblearn, tabgan, ‐and SHAP.

## 3 RESULTS

**FIGURE 1:**
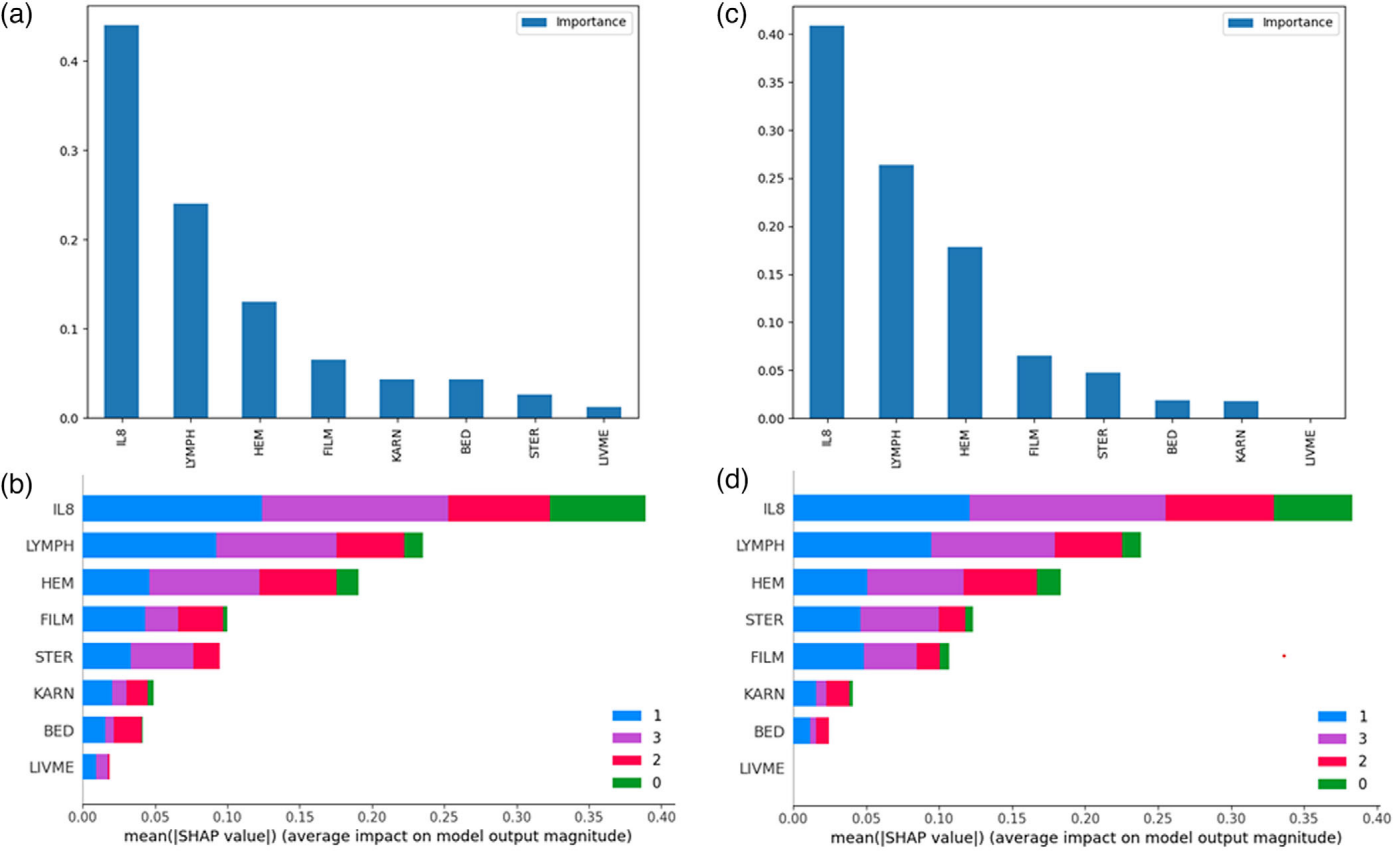
Figures 1a and 1c show the importance ranking percentage of the covariates obtained by the (a) entropy‐based and (c) Gini impurity‐basede decision tree algorithms. Figures 1b and 1d show the SHAP summary plots of the feature value impact on the two‐decision tree multiclass models across all four classes.

573 patients were included in this study. Among them, 32 (5.6%), 219 (38.2%), 127 (22.2%) and 195 (34.0%) belong to CLASS0, CLASS1, CLASS2, and CLASS3, respectively. Missingness was minimal, with all variables (including laboratory parameters) having greater than 90% completeness. After feature dimensionality reduction, 8 features were used in DT modeling. Among these, four clinical variables (use of steroid (STER), presence of liver metastases (LIVME), food intake variation last month (FILM) and Karnofsky index (KARN)), three laboratory variables (interleukin8 (IL8), hemoglobin (HEM) and lymphocytes count (LYMPH)) and one dosimetric variable (biological equivalent dose (BED)) were selected. Figures 1 show the feature importance ranking percentage for the entropy‐based (1a) and Gini‐based models (1c), respectively. This ranking is confirmed by the SHAP importance chart, as reported in Figure 1b and 1d. In these last graphs, the multi‐colored bar for each feature breaks down how much influence a feature has exerted over each of the four possible outcomes for OS. The first three ranked variables are the laboratory variables, namely the interleukin‐8, the lymphocytes count, and the hemoglobin. In particular, SHAP plots show that these three laboratory variables represent the major impact on OS in each of the four classes, accounting for more than 80% of the contribution. Following, with an increasingly less important weight, are the use of steroids and/or food intake last month, the Karnofsy index and the BED.

Table 1 reports the baseline characteristics of the surviving variables in the study population and in the four different classes.

**TABLE 1:** Baseline characteristics of the surviving variables in the study population and in the four different classes.

Variable	Overall (*n* = 573)	CLASS 0	CLASS 1	CLASS 2	CLASS 3	*p*‐value
Interleukin‐8 (IL8, pg/mL)	18.2 ± 25.6	61.0 ± 61.2	21.8 ± 27.4	13.2 ± 8.6	10.3 ± 7.4	< 0.0001
Lymphocites (LYMPH, %)	19.4 ± 10.6	11.9 ± 7.3	16.6 ± 9.5	20.1 ± 9.2	23.4 ± 11.3	< 0.0001
Hemoglobin (HEM, g/dL)	12.3 ± 1.7	10.9 ± 1.5	12.0 ± 1.7	12.1 ± 1.6	13.0 ± 1.6	< 0.0001
Use of steroid (STER)						< 0.0001
Yes	191 (33.3%)	16 (50%)	95 (43.4%)	43 (33.9%)	37 (19.0%)	
No	382 (66.7%)	16 (50%)	124 (56.6%)	84 (66.1%)	158 (81.0%)	
Food intake variation in the last month (FILM)						< 0.0001
Yes	246 (42.9%)	19 (59.4%)	119 (54.4%)	50 (39.3%)	58 (29.7%)	
No	327 (57.1%)	13 (40.6%)	100 (45.6%)	77 (60.7%)	137 (70.3%)	
Karnofsky performance status (KARN)						< 0.0001
< 80	328 (57.2%)	27 (84.4%)	155 (70.8%)	64 (50.4%)	82 (42.1%)	
≥ 80	245 (42.8%)	5 (15.6%)	64 (29.2%)	63 (49.6%)	113 (57.9%)	
Presence of liver mets (LIVME)						< 0.0001
Yes	157 (27.4%)	18 (56.3%)	75 (34.2%)	33 (26.0%)	31 (15.9%)	
No	416 (72.6%)	14 (43.7%)	144 (65.8%)	94 (74.0%)	164 (84.1%)	
Biological equivalent dose (BED, Gy)	26.7 ± 11.0	22.5 ± 9.3	26.3 ± 10.6	25.3 ± 10.9	28.8 ± 11.2	0.001

**FIGURE 2:**
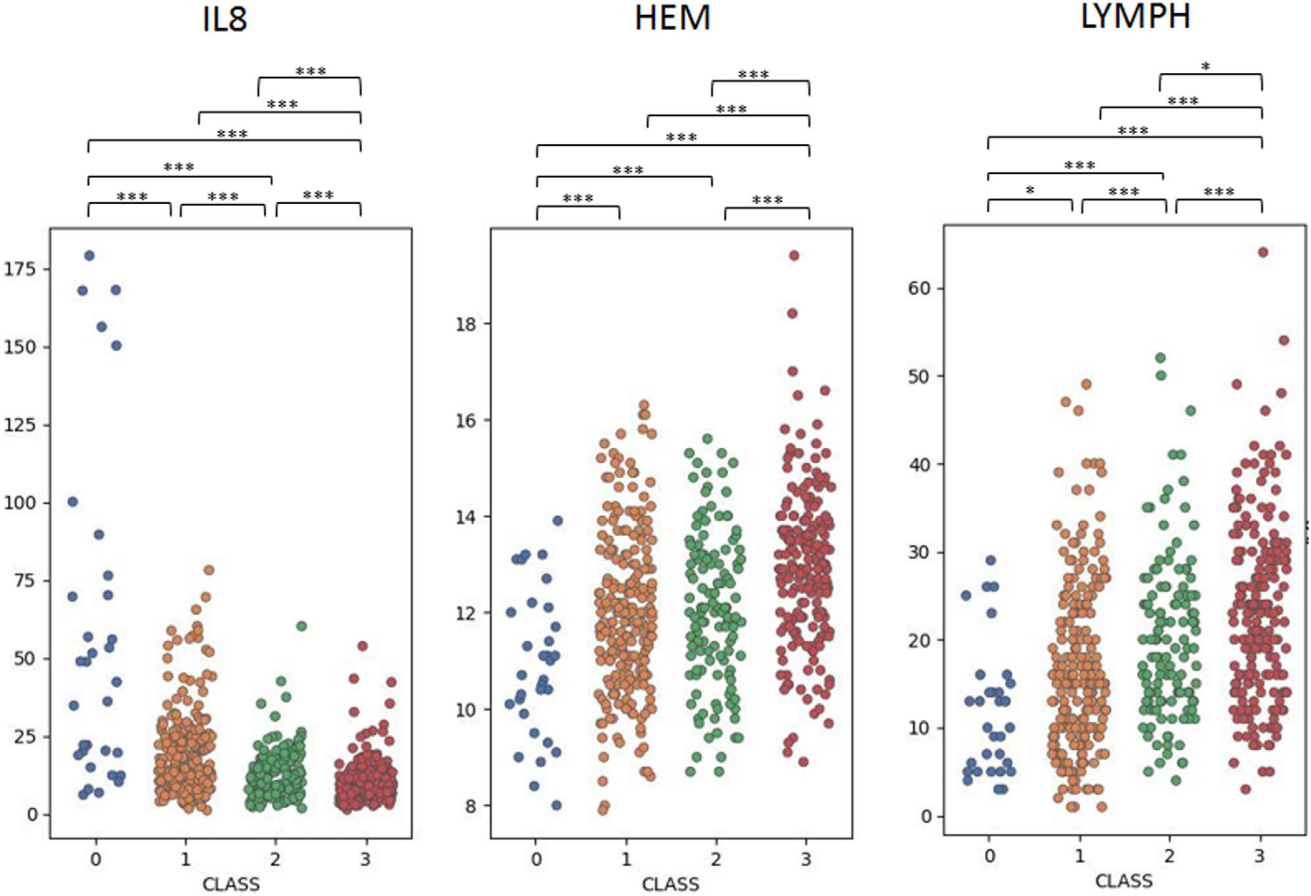
Characteristics of laboratory variables in patients belonging to the four survival classes. *p < 0.05, ***p < 0.001.

Figure 2 shows the distributions of the three most important numerical variables (IL8, LYMPH and HEM) among the four survival classes as strip plots. IL8 displayed significantly higher values in the CLASS 0 compared with the other classes and continued to decrease significantly as survival times increase. On the contrary, both HEM and LYMPH variables reported significant higher values from CLASS 0 to CLASS 3 as survival times increased.

**FIGURE 3:**
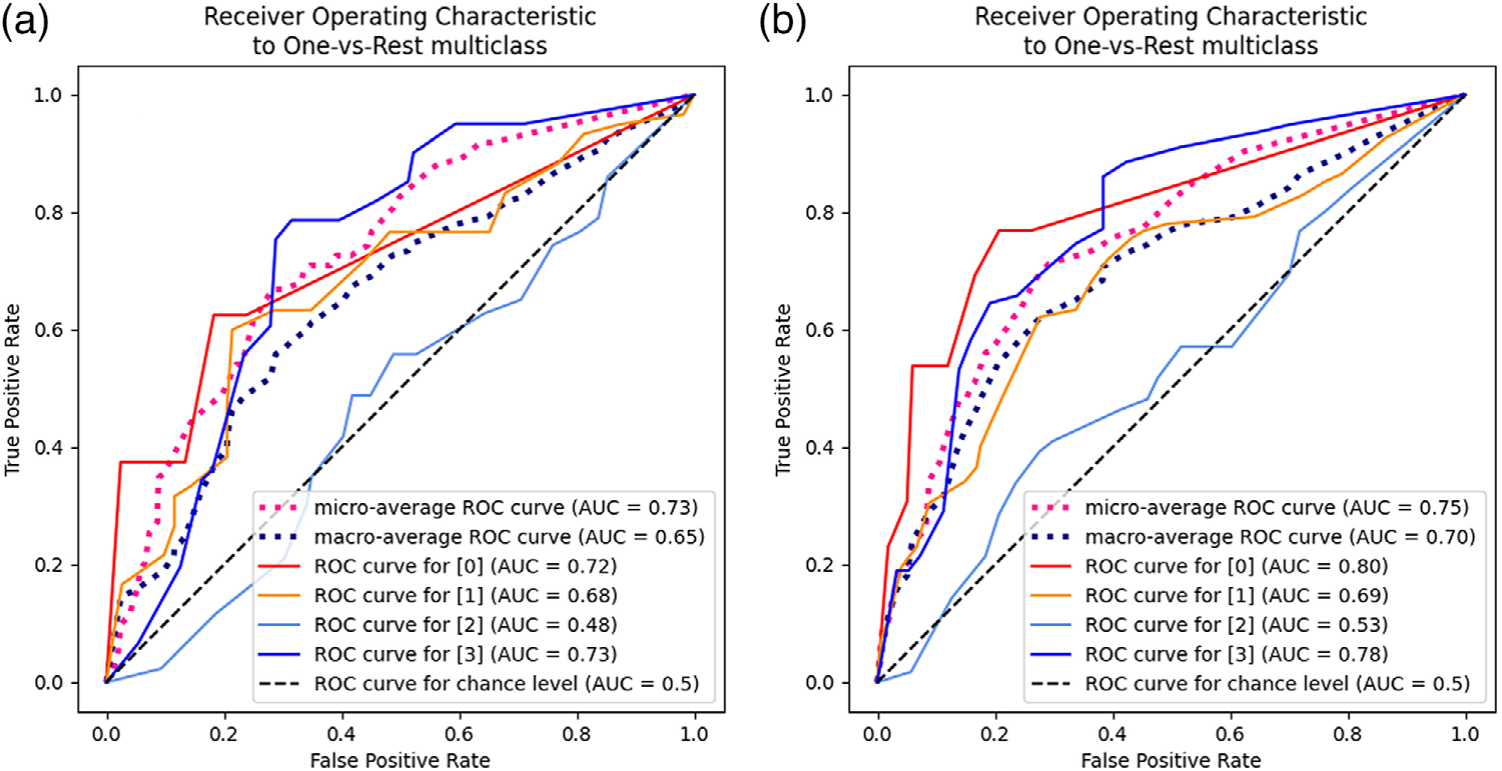
Four‐class (one‐vs‐rest) ROC curves of (a) the entropy‐based model and (b) the Gini‐impurity based model for the testing sets. The two dashed lines show the ROC curves of micro‐ and macro‐average, indicating the overall distinguishing ability of the four‐class classification.

Figure 3 shows the four‐class (one‐vs.‐rest) ROC curves for the (a) entropy‐based and (b) Gini‐impurity based models for the testing sets. The two dashed lines show the ROC curves of micro‐average and macro‐average, indicating the overall distinguishing ability of the four‐class classification. The Gini‐based model performed better than the entropy‐based one; in particular, CLASS 0 and CLASS 3 reported an excellent discrimination ability with the higher AUCs (0.80 and 0.78, respectively). In both models, CLASS 2 reported an AUC value close to 0.5, corresponding to a classification model basically worthless for this class.

**FIGURE 4:**
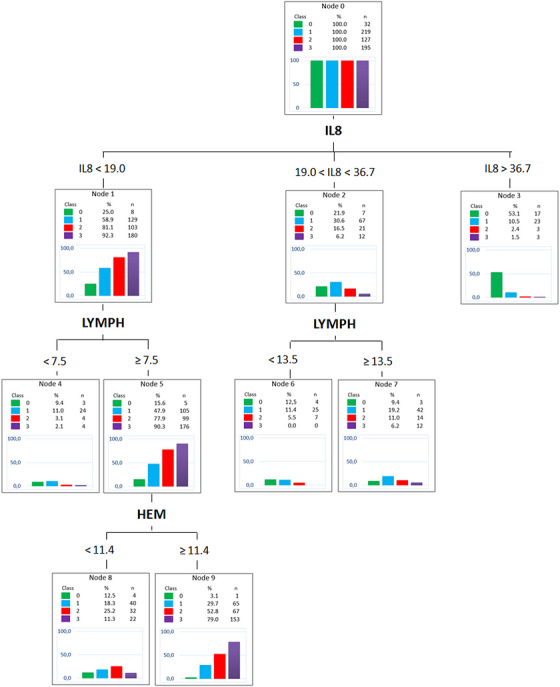
Decision tree for the Gini_based method. IL8 = interleukin8, LYMPH = lymphocyte count and HEM = haemoglobin.

The classification tree for the most informative variables, obtained from the Gini‐based DT model, is displayed in Figure 4. Node 0 illustrates the initial distribution of the four survival classes for the 573 patients. Nodes 1, 2, and 3 show the resulting classification derived by IL8 values as the first criterion for decisions. Patients with pre‐treatment values of IL8 < 19 are relocated to node 1 and patients with 19 < IL8 < 36.7 and IL8 > 36.7 are relocated to node 2 and 3, respectively. Node 3 (IL8 > 36.7) relocated more than 50% of patients with survival < 3 weeks and only 1.5% of patient with survival > 52 weeks. On the other side, node 1 (IL8 < 19) relocated about 92% of patients with survival > 52 weeks and only 25% of patients with survival < 3 weeks. Patients are then additionally separated based on a second criterion, that is, LYMPH. At nodes 4 and 5, LYMPH values higher than 7.5 will drive the probability of survival > 52 weeks still over 90% while it drops down to 2.1% for LYMPH < 7.5. The figure effectively demonstrates the interpretability of a decision tree model, that is, its ability to assist clinicians in comprehending the reasoning behind the choices adopted by the algorithm.

## 4 Discussion

A recent letter published in the Journal of Applied Clinical Medical Physics and titled *“Embracing Real AI: A call to action for medical physicists in healthcare* *15* *”*highlighted the urgency for medical physicists to prepare for the integration of artificial intelligence into healthcare practices and emphasized their pivotal role in adapting to technological advancements. The authors claim for a broadening of perspectives, encouraging interdisciplinary collaborations with professionals from other disciplines such as computer science, data science, and biomedical engineering. This will enable medical physicists to provide continuing education and connect the community with research opportunities. These new challenges will require medical physicists to continuously update skills and innovate education, adapt curricula to include new fields, and reinforce multidisciplinary attitude and spirit of innovation.

In line with this invitation, in this paper we investigated the performance of decision tree models in the multiclass classification of OS in patients with bone metastases following PRT. Our goal was to provide an accurate life expectancy prediction for patients with advanced cancer, which should lead to better treatment decisions. This is an ambitious project but worthy of every effort, since appropriate palliative care is associated with improved patient quality of life.^16^ To the best of our knowledge, the current analysis is one of the most comprehensive in this clinical setting, with the added benefit of incorporating a large amount of laboratory data in addition to parameters related to patients, tumors, and treatment characteristics.

The developed DT classifiers demonstrated the potential to ascertain the patient's risk of death at various time endpoints before receiving PRT. In particular, the multiclass analysis provided higher performances for patients risk of death at short‐term (< 3 weeks) and long‐term (> 52 weeks). This is especially important because a patient with advanced cancer dying within a short interval before the beginning of a treatment is a more complex and hazardous scenario than that of a patient with a longer survival interval. In particular, in patients with very short survival, best supportive care is often preferred over palliative treatments (e.g., PRT), as the latter can cause discomfort in those with advanced disease and may not be completed or have sufficient time to be effective.^17^ On the other hand, the observed AUC values close to 0.5 for the class 2 (OS: 24–52 weeks) indicated that the model provided no discriminative advantage over random chance, as the true positive rate is roughly equal to the false positive rate across all thresholds. The poor performance for this class suggests that the several variables analyzed may not fully account for the multi‐factorial nature of overall survival in this patient cohort. These findings highlight the inherent challenges in modeling survival outcomes and emphasize the need for more complex, non‐linear approaches or the integration of novel biomarkers.

In the meantime, we showed that DT algorithms provide an intrinsic explainability approach to machine learning, are able to highlight the salient characteristics of the model, and are easily understood by clinicians thanks to the hierarchical tree visualization.

The analysis reported interesting results, including the secondary role of traditionally considered clinical prognostic factors (i.e. Karnofsky index or the use of steroid). On the contrary, a few laboratory data such as IL8, LYMPH and HEM turned out to be the strong predictors of patient survival at different time endpoints. As reported in Figures 2 and 4, the model's predictions are driven toward high chance of survival at different times by low IL8 values (IL8 < 19) and high hemoglobin and lymphocyte count value. While it is expected that one‐year survival should be more influenced by factors related to the progression of the cancer disease (i.e. the role of IL8), a major unexpected finding of this study is that also short‐term survival is more related to the laboratory variables and not to patient performance status, as might be expected. As first decision criterion, our resulting tree analysis chose the interleukin‐8 variable to be strongly associated with OS for all survival time endpoints. In particular, more than 50% and 98% of patients with high IL8 values (IL8 > 36) are strongly associated with short‐term (OS < 3 weeks) and long‐term (OS > 52 weeks) mortality, respectively. Subsequently, the lymphocytes count and, with less importance, the hemoglobin values played a significant role in increasing the classification rate among the patients. This tree model may be a useful and valuable tool for radio‐oncologists to customize efficient treatment in a multidisciplinary context.

The role of these three laboratory parameters in cancer prognosis is continuously emerging in the recent literature. Nowadays, the value of IL8 in the peripheral blood represents a well‐established prognostic marker in different types of cancer.^18^ Higher levels of IL8 have been linked to worse prognoses for pancreatic and ovarian cancer.^19^, ^20^ Moreover, in metastatic breast cancer, a recent trial reported a strong association between low survival rate and high levels of IL8 (> 16.6 pg/mL), with a median value that dropped from 47.5 to 20.5 months. Similarly, a few studies highlighted the role of anemia as a driving parameter to poor local control and survival rates, especially in head‐and‐neck cancer^21^, ^22^ and cancers of the uterine cervix.^23^ It has been shown that anemia reduces cell oxygenation and increases resistance to chemoradiation by oxygen deprivation, which is a crucial part of the cytotoxic action of these treatments.^24^ Lastly, also low lymphocyte count also has been associated with poor outcomes in advanced cancer patients receiving palliative therapy.^25^ Our results are consistent with the aforementioned literature. Because these laboratory parameters should be considered crucial, clinicians should be aware of the significance of these variables in predicting the survival of patients treated with PRT.

In our opinion, the present study marks three important contributions. Firstly, it stands as one of the first to use machine learning modeling for a classification approach to predict survival in patients with advanced cancer following PRT in a real‐world large dataset including also laboratory data. If a number of validated prognostic instruments have been developed to predict the survival of patients with advanced cancer,^26^, ^27^, ^28^, ^29^ however, the use of subjective variables, such as the patient's symptoms and condition, represents a significant drawback due to clinician experience and skill level.^30^ As a result, it has been highly advised to employ objective variables—such as laboratory results and vital signs—more extensively when creating prognostic models.^31^ In the field of PRT, the value of laboratory data in predicting survival in cancer patient's is an emerging field of research. A recent paper demonstrated the clinical impact of selective laboratory values such as serum albumin level, hemoglobin level, and lymphocyte count on survival prediction obtained by machine learning in patients receiving radiotherapy for spinal metastases.^32^ Secondly, departing from binary classification, we introduced and utilized four clinically meaningful classes for survival analysis. This approach aims for more accurate predictions, estimating the anticipated length of survival to enable accurate and efficient planning for palliative care. Lastly, the study focused on decision trees to interpret survival predictions in order to increase transparency and enhance its reliability and robustness. Decision trees demonstrated a major versatility in simulating intricate decision‐making processes because of their interpretability and versatility. They are skilled with both numerical and categorical data, and because of their independent feature selection ability, they can readily adapt to a wide range of datasets. As reported in Figure 4, they provide simple visualization, which helps to comprehend and elucidate the underlying decision processes in the model. Unlike complex models like deep neural networks, they have a straightforward, rule‐based structure, allowing for a clear hierarchy of decision importance. This remains a key point toward the necessary steps in building confidence in the utilization of machine learning models in clinical decision‐making.

A few potential limitations of the present study need to be addressed. First, despite the use of advanced ML models, the limitations of retrospective analyses remain. Furthermore, despite the fact that this was a large multicenter study, the majority of the patient population was Caucasian and came from European centers, which may have limited the applicability of the findings to other geographic and ethnic contexts. Furthermore, our study lacked external validation from a different cohort, which could have confirmed our model's performance. In palliative radiotherapy, the choice of fractionation and assessment of pain response can vary significantly between Centers. The lack of external validation prevents us from confirming whether the model maintains its accuracy even in settings with different treatment philosophies or populations with different metastatic burdens, currently limiting its clinical scalability. At the moment, our results should be framed as hypothesis‐generating rather than ready for routine clinical use. Future efforts will be directed at validating the model on independent cohorts, possibly through international multicenter studies that reflect different real‐world clinical practices.

Moreover, future research could focus on developing software or applications that oncologists could use in real‐time consultations to assist with personalized treatment decisions. These tools could enhance clinical practice by integrating the decision‐tree models into routine care, allowing oncologists to make data‐driven adjustments to treatment and palliative strategies based on predicted survival outcomes. Additionally, incorporating other data types, such as genomic information, could improve the model sensitivity and accuracy. Specific studies are needed to explore how these models could be practically applied in clinical decision‐making and whether they would influence radiation oncologists' approach to care. Finally, given the sensitive nature of palliative care and life expectancy predictions, ethical considerations must be addressed. Future research should investigate how clinicians can balance machine‐generated predictions with patient preferences, particularly in scenarios involving short‐term survival predictions.

## 5 Conclusion

We reported that ML decision tree models for risk mortality were able to predict the survival at different timing in patients with advanced cancer who underwent PRT for bone metastasis. These models provided an intelligible explanation of individualized risk prediction, helping clinicians to better understand the decision‐making process involved in the evaluation of overall survival.

## AUTHOR CONTRIBUTIONS

All authors have contributed to the work. Each author did have substantial contributions, drafted/reviewed the manuscript and approved the final version.

## CONFLICT OF INTEREST STATEMENT

The authors declare no conflicts of interest

## ETHICAL APPROVAL

The study was conducted according to the guidelines of the Declaration of Helsinki and approved by the Area Vasta Romagna Ethics Committee (code: L2P1517; May 17, 2017).

## Supporting information



TABLE S1 Overall overview of the collected variables

## Data Availability

Research data are stored in an institutional repository and will be shared upon request to the corresponding author.
