# Supplementary material for: Machine learning decision tree models for multiclass classification of prognosis in patient undergoing palliative radiotherapy for bone metastases
Source: J Appl Clin Med Phys. 2026 Jul 8;27(7):e70606. doi: 10.1002/acm2.70606 (PMC13345205; doi:10.1002/acm2.70606)
Supplement: Supplementary file 1 — TABLE S1 Overall overview of the collected variables [file ACM2-27-e70606-s001.docx]

Table S1

*Clinical variables*

Gender

Weight

Height

Body mass index

Karnofsky Performance Status

Weight variation of the Last 2 Weeks

Food Intake variation in the last Month

*Tumors-related variables*

Liver Metastases (Yes/No)

CNS Metastases (Yes/No)

Lung Metastases (Yes/No)

Other Metastases (Yes/No)

Extension of bone metastases to soft tissues (Yes/No)

Osteolitic bone metastases (Yes/No)

*Dosimetric and treatment-related variables*

Number of fractions

Dose per fraction

Reirradiation (Yes/No)

Chemotherapy (Previous/Concurrent)

Hormonal Therapy (Previous/Concurrent)

Opiods (Yes/No)

Steroids (Yes/No)

*Laboratory variables*

Hemoglobin

White blood cells

Lymphocites

Neutrophils

Monocites

Platelets

C reactive protein

Creatinine

Urea

Albumin

Bilirububine

Potassium

Sodium

Chloride

Calcium

Phosphorus

Magnesium

Triglycerides

Vitamin D

TNF-alpha inhibitors

Interferon gamma levels (INF-γ)

Interleukin 1 beta (IL-1B)

IL-1 Receptor Antagonist (IL-1Ra)

Interleukin-2 (IL-2)

Interleukin-4 (IL-4)

Interleukin-5 (IL-5)

Interleukin-6 (IL-6)

Interleukin-7 (IL-7)

Interleukin-8 (IL-8)

Interleukin-9 (IL-9)

Interleukin-10 (IL-10)

Interleukin 12 p70 (IL-12 p70)

Interleukin-13 (IL-13)

Interleukin-15 (IL-15)

Interleukin 17A (IL-17A)

Monocyte Chemotactic Activating Factor (MCP-1/MCAF)

Interferon-γ–Inducible Protein 10 (IP-10)

Eotaxin

Macrophage Inflammatory Proteins 1A (MIP1A)

Macrophage Inflammatory Proteins 1B (MIP1B)

Regulated on activation, normal T cell expressed and secreted (RANTES)

Granulocyte colony stimulating factor (G-CSF)

granulocyte macrophage-colony stimulating factor (GM-CSF)

Basic Fibroblast Growth Factor (BFGF)

platelet-derived growth factor (PDGF-BB)
